# The Justice and Ontology of Gastrospaces

**DOI:** 10.1007/s10677-022-10357-x

**Published:** 2023-01-27

**Authors:** Matteo Bonotti, Andrea Borghini, Nicola Piras, Beatrice Serini

**Affiliations:** 1 Monash University, Melbourne, Australia; 2 University of Milan, Milan, Italy

**Keywords:** Food and space, Justice, Ontology, Moral powers

## Abstract

In this paper, we establish gastrospaces as a subject of philosophical inquiry and an item for policy agendas. We first explain their political value, as key sites where members of liberal democratic societies can develop the capacity for a sense of justice and the capacity to form, revise, and pursue a conception of the good. Integrating political philosophy with analytic ontology, we then unfold a theoretical framework for gastrospaces: first, we show the limits of the concept of “third place;” second, we lay out the foundations for an ontological model of gastrospaces; third, we introduce five features of gastrospaces that connect their ontology with their political value and with the realization of justice goals. We conclude by briefly illustrating three potential levels of intervention concerning the design, use, and modification of gastrospaces: institutions, keepers, and users.

*- A party without cake is just a meeting.*

Julia Child

## Introduction

Restaurants, home kitchens, cafes, pubs, dining tables, takeout places, food trucks, food street vendors, markets, ice cream parlors, picnic areas, beaches, public squares, waiting rooms, gardens, backyards, cars, buses, trains, airplanes… This is only a short list of sites that human beings, nowadays and throughout history, have been using as spaces to eat and drink (Rawson & Shore 2019: 9–29). It is a list that evokes a complex history of eating practices,^1^ more or less intentionally designed to accompany people’s everyday needs, toils, and leisures. We shall refer to these spaces with the expression *gastrospaces*, a neologism stressing the close ties between food and space.

In this paper, we set out to establish gastrospaces as a subject of scholarly inquiry as well as an item to include in policy agendas at local and non-local levels. We maintain that such spaces can be key sites of justice and injustice. The core of our argument points at their capacity to enable or hinder people’s ability to develop and exercise two fundamental moral powers, what John Rawls calls the “capacity for a sense of justice” and “the capacity to form, to revise, and rationally to pursue a conception of one’s rational advantage or good” (Rawls 2005: 19). For example, at the outset of her study on the importance of “table talk” for democratic agency and participation, Flammang (2016: 2) observes:

Eating is something we do frequently […]. Of course, there are cultural differences in rules about civil tables—who should speak, when and how; what behavior is acceptable; what topics are off limits; and how conflict should be resolved. Indeed, it is our daily exposure to the making, enforcing, and breaking of these rules that constitutes our daily doses of political awareness, growth, and transformation. It is at tables and in conversations that we make sense of the many layers of our experiences with political import.

Here we take a broader perspective than Flammang, by considering not only the conversational and “civil” dimensions of gastrospaces, but also the political issues surrounding the distinctively *spatial* dimensions of these sites—such as urban planning, design, access, and inclusion/exclusion—which raise important questions of justice.

Our approach combines the resources of analytical political philosophy and analytic ontology. On the one hand, it brings out the conceptual and ontological assumptions that implicitly underlie much political philosophical analysis; we focus specifically on the properties of gastrospaces to better understand where, when, and how it is possible to intervene in order to more effectively realize justice goals in such spaces. On the other hand, our approach employs political philosophy to develop a theoretical understanding of gastrospaces that is driven by (and aimed at realizing) specific justice goals, and which can help devise real-world interventions. Our aim is to provide a philosophical account of those entities that we think should be of special significance in public and academic discourse.

We begin (§2) by explaining what the value of gastrospaces is in relation to the development and exercise of the two moral powers, thus clarifying the relevance of our topic from the point of view of political philosophy.^2^ This analysis, however, would be incomplete without a proper account of what gastrospaces are. More specifically, it would lack a clear understanding of the key components of such spaces, and of how this information could be used to design or modify them in order to advance the two moral powers. To this effect, in §3 we unfold a theoretical framework for gastrospaces, in three steps: first (§3.1), we show the limits of the concept of “third place;” second (§3.2), we lay out the foundations for an ontological model of gastrospaces, which maps out the most basic entities included within them; third (§3.3), we introduce five features of gastrospaces that integrate our ontological model and can help us to better understand whether and how the ontological properties of such spaces are linked to the development, exercise, and realization of the two moral powers.^3^ As well as being theoretically innovative, our study can also provide a novel blueprint for real-world interventions regarding gastrospaces. We conclude (§4) by briefly illustrating how three potential categories of actors can benefit from our framework in relation to the design, use, and modification of gastrospaces: institutions, keepers, and users.

## Justice and Gastrospaces

The importance of gastrospaces in shaping and cementing social relationships in contemporary societies is well known. The COVID-19 pandemic offers a clear example: widespread lockdowns in many countries forced the temporary closure of restaurants, cafes, and other eating spaces, thereby precluding the conditions for social gatherings (Bonotti et al. 2022; Bonotti and Zech 2021) and depriving many people of the opportunity to share significant meals and to engage in social interactions (e.g., see Ammar et al. 2020). Meanwhile, those restrictive measures also changed the way people ate at home and contributed to the development of new foodways such as digital commensality.^4^ Yet, despite their importance, gastrospaces have not been systematically examined within normative political philosophy: What is their role in a theory of justice? What kinds of moral goals do they help to realize?

To address these and related questions we take a broadly Rawlsian perspective grounded in Rawls’s influential account of the two “moral powers:” “the capacity for a sense of justice” and “the capacity to form, to revise, and rationally to pursue a conception of one’s rational advantage or good” (2005: 19). In order to be able to develop and exercise their two moral powers, all individuals need access to certain primary goods, i.e., “all-purpose means,” which include basic rights and liberties, the opportunity to access positions of power and responsibility, income and wealth, and the social bases of self-respect. The latter provides each person with “a…sense of his own value, his secure conviction that his conception of his good, his plan of life, is worth carrying out…[as well as]…a confidence in one’s ability, so far as it is within one’s power, to fulfill one’s intentions” (Rawls 1999: 386).

Our choice to focus on the two Rawlsian moral powers rests on their marked “thinness” and flexibility: all individuals possess them regardless of their conception of the good and their legal status (e.g., regardless of whether they are legal citizens or resident non-citizens).^5^ These aspects (i.e., thinness and flexibility) also afford us key theoretical flexibility: they ensure that our analysis also applies to transient or “invisible” individuals, such as travelers and illegal residents.^6^

On what grounds, then, can we argue that gastrospaces are a legitimate concern of theories of justice? To answer this question, we follow Cordelli (2015: 94). In her analysis of justice and relational resources, she suggests three criteria for establishing whether a good should be considered as primary and, therefore, a concern of theories of justice:

1. The good should be “generally necessary [although not sufficient] for the development and exercise of (at least one of) the two moral powers.”
2. The good should be “valuable across a variety of conceptions of the good, without their value being grounded in any such conception.”
3. Social institutions should play a role in the distribution of the good.

In what follows, we set out to show that gastrospaces meet each of the three criteria.^7^ We begin by considering the first criterion in connection with both moral powers, starting from the second of such powers.

### The First Criterion: Gastrospaces and the Two Moral Powers

Gastrospaces are crucial for individuals’ ability to exercise their second moral power, i.e. to realize their life plans and conceptions of the good, insofar as they provide key sites where to prepare or consume food, both at home and in public. For example, access to certain gastrospaces, such as soup kitchens (e.g., see Marovelli 2019), serves to realize food security, a basic necessity for any individual to be able to pursue their conception of the good, whatever the latter might be. Likewise, other gastrospaces, like home kitchens, are not only an effective means for guaranteeing the right to cook one’s own food, but also spaces where individuals and groups gain or even reconstruct a sense of stability and belonging (Supski 2006; Longhurst et al. 2009), or foster collective memories and identities rooted in cultural and familiar practices (Meah and Jackson 2016). In this sense, gastrospaces can promote individuals’ *inherently* valuable relationships and goals as is also the case, for example, with mosque and synagogue canteens serving halal and kosher meals, which provide Muslims and Jews with access to food that complies with norms central to their religious faiths and cultural identities (Barnhill et al. 2014).^8^ Or they can enable self-expression and creativity,^9^ leisure, or the aesthetic appreciation of food. In other cases, gastrospaces can help realize *instrumentally* valuable goals and relationships, e.g., by advancing people’s work opportunities^10^ or providing them with access to networks of trust, care, emotional support, or social influence (cf. Cordelli 2015: 94–96).

Gastrospaces can also provide individuals with the social bases of self-respect. When eating out is a widespread practice in a society, for example, not being able to enjoy it can seriously undermine a person’s self-respect^11^—think, for example, of a child who can never eat the food linked to their culinary traditions outside their home because it is either not available anywhere or because consuming it is frowned upon even by acquaintances and friends. Or consider how refugee camps not equipped with sufficient cooking technologies can have a negative impact on the self-respect of those who live there (see, e.g., Barbieri et al. 2017 who offer an overview of cooking facilities in refugee camps).

Let us now consider the first moral power, i.e., the capacity for a sense of justice, and assess whether and how gastrospaces can help enhance it: how can gastrospaces help people understand and act according to the moral duties they owe to each other? Restaurants that advance a conception of justice centered on animal welfare by only serving vegan or vegetarian food,^12^ or those that charge different prices for the same meal based on customers’ income,^13^ are instances of gastrospaces that can help individuals to act in ways that further what they consider justice goals. For example, they can help cement bonds between fellow vegans or vegetarians, or between those who want to fight socio-economic inequalities, and potentially encourage other people to also pursue those justice goals. Consider, also, the role played by certain gastrospaces in promoting gender equality—e.g., feminist restaurants, cafes, and coffeehouses in Canada and the United States during the 1970s and 1980s, which contributed to advancing women’s empowerment against male oppression (Ketchum 2018), or gay bars that help foster LGBTIQ + people’s equal rights and recognition (Sisson 2016). Other kinds of gastrospaces, which are located in a gray area between public and private spheres—e.g., shared kitchens—might instead strengthen social ties between different members of the same society while developing “a sense of community around food” (see, e.g., the Australian project *Community Kitchens*).^14^

The first moral power is also advanced by the development of relationships of trust among those who frequent gastrospaces, especially when such spaces further inclusive experiences (e.g., a public park where different groups can share cooking equipment and food), rather than being the expression of one or a few dominant identities and interests. These kinds of intergroup relationships can help reduce prejudice and discrimination across society, thus contributing to justice in an important way (cf. Allport 1954; Pettigrew 1998).

Finally, gastrospaces can help individuals to access knowledge that may be central to the advancement of justice goals. Eating out in certain spaces can provide individuals with important information related to justice issues (Gopnik 2018) and it can make people more aware of the society that surrounds them, offering insights on its social landscape and how it may change over time (Gibson and Molz 2016: 84–85). For instance, White (2012: 138) describes the role of *kissaten* (Japanese cafès) as “transitional facilitators” for those “migrant workers from the countryside, needing to learn the urban ropes.” Gastrospaces can also become important settings for debate and exchange of ideas, enhancing democratic skills that are often central to the advancement of justice goals. Consider, for example, the role of the local cafe as a site of political debate (see the *locus classicus* Habermas 1989) but also the dining table (including at the domestic level) as a stage for integration or conflict (Flammang 2016).

In summary, then, gastrospaces can often be places where people can live typical relations as citizens, where the latter term should be understood broadly in a non-legal sense to also include non-citizen residents as well as transient or “invisible” individuals. In gastrospaces, we can meet people who are very different from or very similar to us; discuss various types of issues; and, more generally, live a (partially) public life. Yet, we should be careful not to reduce the role of gastrospaces to this function. As we explained earlier, gastrospaces can often also foster the exercise of the second moral power, which is normally tied to people’s non-public identities, values, and allegiances rather than to their role and relations as citizens.

At this point, one might observe that while gastrospaces may be *conducive* to the exercise and development of the two moral powers, they are in fact not *necessary* for them. Individuals, that is, can at least in principle cultivate their conceptions of the good and advance their justice goals independently of gastrospaces. However, this conclusion seems to neglect some important facts about contemporary societies.

First, when access to certain kinds of goods is pervasive and intertwined with most people’s everyday lives, it is difficult to argue that such access is not an issue of justice. Take, for example, access to the Internet or to some form of transportation. Since work, educational and relational opportunities are nowadays inextricably dependent on people’s ability to have access to the Internet and to some kind of transportation, it would be unfeasible to argue that these are not primary goods essential for the exercise of our two moral powers. Likewise, gastrospaces are intertwined with most, if not all, people’s everyday lives. Workplace, school and university canteens, restaurants, cafes, pubs, etc. permeate and play a central role in people’s lives in contemporary societies (Warde and Martens 2000). Furthermore, in many societies key life events such as children’s birthday parties, weddings and, sometimes, funerals are typically held in gastrospaces, e.g., restaurants, cafes or people’s homes. Given all of that, if and when gastrospaces are, for example, inaccessible to certain types of people (e.g., because of their design, location, etc.), it would be puzzling to argue that this is not an issue of justice.

Second, and relatedly, it is not always possible for people to reproduce outside certain gastrospaces the kinds of experiences that the latter enable, and which help people to exercise their moral powers. In fact, some gastrospaces are particularly apt to foster some special social relationships precisely because they provide a neutral and non-familiar environment. Examples include business negotiations, political meetings, and romantic dating (for a sociological analysis of the role of eating out in those occasions, see Warde and Martens 2000, in particular chapters 9 and 10).

Third, we should be careful not to reduce gastrospaces to the narrower category of spaces of eating *out*. While the latter, as we have just argued, are nearly ubiquitous in most contemporary societies, the exercise and development of the two moral powers can also occur in a wide spectrum of domestic and seemingly more private gastrospaces, thus further demonstrating the latter’s pervasiveness. Think, for instance, of how the design of domestic spaces intersects with the history of women’s emancipation. A well-known case is the so-called ‘Frankfurt kitchen.” Designed by the Austrian architect Margarete Schütte-Lihotzky as a means to female emancipation, the kitchen stood out for its innovative spatial arrangement and equipment, aimed at economizing time and labor, hence eliminating domestic drudgery (Henderson 1996; Hessler 2009). Conceived to foster the demands of the German feminist movement, however, the Frankfurt kitchen eventually came to incarnate the “female redomestication” process in the Weimar Republic, forging a “professional workplace” for women that eliminated the “urge” to emancipate by leaving their homes. This example bears witness to the political importance of domestic gastrospaces—in addition to spaces of eating out—as sites of justice and injustice.

In sum, therefore, gastrospaces—whether public or private—are inherently intertwined with our daily lives and, therefore, necessary for the exercise of our moral powers.

### The Second Criterion: The Neutral Value of Gastrospaces

While gastrospaces, we have seen, are necessary for the exercise of our moral powers, are they also “valuable across a variety of conceptions of the good, without their value being grounded in any such a conception”? It is evident, from the many examples that we have already provided, that gastrospaces are valuable for different people due to different reasons, and that therefore they are not linked to any specific conception of the good. Whether someone is a vegan or a carnivore, a religious person or an atheist, a conservative or a progressive, access to gastrospaces can be important for the development and exercise of either or both of their moral powers. We are not claiming, however, that *every* particular gastrospace can nurture *every* conception of the good. Inevitably, some of these spaces are closer than others to a specific (set of) conception(s) of the good whereas others are more flexible. Our key point, instead, is that as a general category gastrospaces do seem to be “valuable across a variety of conceptions of the good,” something that arguably could not be said regarding other categories of spaces, such as places of worship, sports centers, or music venues.

### The Third Criterion: Gastrospaces and Social Institutions

Finally, we need to assess whether social institutions play a role in the distribution of gastrospaces as social primary goods.^15^ It seems evident that gastrospaces depend on social institutions in a variety of forms and grades, from less (e.g., accidental gastrospaces during a walk) to more institutionalized ones (e.g., restaurants and cafes). There are, more specifically, two kinds of institutions that we believe play a role in the distribution of gastrospaces as social primary goods: the first comprises *legal institutions* that regulate many aspects of the design, organization, and management of such spaces—e.g., floor plan requirements and opening hours; rules establishing whether food or drink can be consumed in a park or on a beach, or what kind of food or drink can be served in restaurants and other venues; or, at the domestic level, kitchen appliance regulations. The second includes *social institutions and norms*—e.g., norms that stigmatize eating or drinking in places of worship; those which frown upon eating out with one’s children (or eating certain foods) after a certain time of the day; or religious norms that mandate certain kinds of kitchen design, such as the kosher requirement in Judaism that kitchens should have two separate sets of utensils, stoves and refrigerators, one for meat and poultry and the other for dairy foods. Some of these norms may be particularly unfavorable for certain people, as they may constrain their ability to develop and exercise their moral powers, especially when their “temporal autonomy” (Cordelli 2015: 103) is limited—e.g., if one always has to work during those hours when most eating out establishments are open then they will have fewer opportunities to eat out than those who have a more flexible working schedule^16^; or if a Jewish family that migrates to a new city is unable to buy or rent a property provided with a kosher kitchen, its members will be unable to pursue their religious conception of the good within their domestic gastrospace without incurring significant financial and/or practical costs.

It seems therefore clear from the foregoing analysis that gastrospaces should be considered primary goods and legitimate concern of justice: given their pervasive presence in contemporary societies, and the myriad of ways in which they are intertwined with people’s everyday lives—both at the domestic level and in public—they are generally necessary for people’s ability to develop and exercise their moral powers; also, as a category, they are not grounded in any specific conception of the good but rather help promote a wide array of such conceptions; and, finally, their distribution depends on a variety of legal and social institutions. Yet, how are we to assess to what extent gastrospaces foster or hinder the development and exercise of people’s moral powers? Which features of such spaces are key for carrying out this kind of evaluation and, where necessary, for modifying and re-designing such spaces? What are the entities that populate such spaces, and how are they related to each other?

While political philosophy can provide us with useful evaluative norms and parameters for assessing the normative importance of gastrospaces, it cannot provide us with a more specific understanding of how such spaces *are*, including their components, agents, individuation, and identity conditions. This additional information is important to better understand where, when, and how to intervene in order to render gastrospaces more conducive to the development and exercise of the two moral powers, e.g., how to design or modify (aspects of) such spaces.^17^ To this end, in the next section we shall integrate the analysis conducted so far with a conceptual framework for representing in a systematic way gastrospaces, including those features of such spaces that are most relevant to people’s development and exercise of the two moral powers.

## Gastrospaces: A Conceptual Framework

### Gastrospaces Beyond Third Places

An obvious starting point for modeling “gastrospaces” suggested in the literature^18^ is by means of the cognate concept of “third places.” Third places are those that constitute a third alternative to home (“first” places) and workplace (“second” places), and that are often viewed as the paradigmatic sites of “eating out” experiences (e.g., Warde et al. 2019). A classic and standard study of third places^19^ is that developed by Oldenburg (1999: 20–42), for whom a third place is any space that presents the following eight features: (1) it is a *neutral ground*, i.e., it can accommodate a wide spectrum of people and social activities; (2) it is a *leveler*, i.e., class and rank are temporarily set aside in this kind of space; (3) it is a *conversation-facilitator*, i.e., “the talk there is good”; (4) it is *accessible*, i.e., in terms of hours and location; (5) it has its own *regulars*; (6) it enjoys a *low profile*, characterized by homeliness and plainness as well as lack of elegance; (7) it is *playful*, i.e., it facilitates different sorts of conversations; and, finally, it is *a home away from home* allowing guests to be in control of their activities and time schedule.

To what extent can we adapt Oldenburg’s analysis of third places to our study of gastrospaces? At first sight, the overlap between the two categories seems evident. And even though third places generally only include paradigmatic sites of eating out such as cafes and restaurants, one could aim to broaden the scope of that category to include *all* places of eating out or away from home, as actions that unfold in a public sphere.^20^ In this sense, one may suggest that “eating out” can stand not only for those practices of consuming food or beverage at establishments such as restaurants, cafes, or pubs, but also for those practices that involve consuming them in “open” venues such as a beach, a park, or a square (e.g., Jacobs and Scholliers 2003; Burnett 2004); in addition, “eating out” may on occasion refer to practices such as eating a special meal at someone else’s home, consuming food or beverages during a trip (e.g., a car or a train ride), or in a casual spot (e.g., during a hike on a mountain). But even stretching the idea of third place to include all spaces of eating out or away from home would not deliver a theoretical notion that can account for the political and justice-related value of all the spaces where we eat.^21^

First, the notion of third place suffers from several defects that hinder its theoretical neutrality. For example, contra (1), some politically aligned cafes do not offer a neutral ground (e.g., Wexler & Oberlander 2017) or, contra (2) and (6), exclusive restaurants are not levelers nor have a low profile (Rawson and Shore 2019: 51–86). Furthermore, some gastrospaces may not be very playful (e.g., traditional ramen shops, where the conversation is discouraged)^22^ whereas others may be hard to reach and therefore not very accessible. Second, the notion of “third place” forces us to draw a distinction between first, second, and third places that is often virtually impossible to establish. More importantly, that distinction fails to capture—and somehow implicitly dismisses—the political value of first- and second-place gastrospaces, i.e., those located at home (e.g., kitchens and backyards) or in the workplace (e.g., canteens), an exclusion that seems arbitrary at the least.

In sum, Oldenburg’s framework—spelled out in terms of allegedly *necessary* features of third places—falls short of providing an adequate conceptual analysis of gastrospaces, as the latter represent a much wider and internally diverse category that can be hardly constrained by one narrow set of clauses. On this note, it is useful to remark that the term “gastro*space*” makes reference to space, and not place, precisely to leave open the possibility that the very same space may be regarded as holding different places from the perspective of different agents.

### Outline of an Ontological Model

We have shown that certain gastrospaces cannot be identified with third places, the latter being a much narrower category. What is required for a systematic analysis of gastrospaces, therefore, is a broader conceptual framework, which includes third places as a special case but also applies to a wider range of cases. The approach we shall propose employs the tools of analytic ontology to develop such a framework. We proceed in two steps: first, we offer a general ontological model of gastrospaces (from which intended models of specific gastrospaces can be derived); second, we use such a general model to track down the key features of gastrospaces that are linked to their justice-related functions.

In general terms, an *ontological model* is a representation of a given domain of entities, both particular (e.g., a particular person) and universal (e.g., the property of being human), along with the relations between them; also included in the representation are the norms regulating the conditions for an entity to be a component of the domain (Arp et al. 2015: 1–27). Ontological models serve as a tools for storing and sharing all the relevant information on a given domain of reality as well as for rethinking it, by categorizing its most salient entities and imagining new possibilities for them: in fact, an ontology depicts not only how the world is but also a space of possibility for it. Such models may be either formal—i.e., they are written in mathematical languages by using standard notations (e.g., logical connectives and formation rules)^23^—or informal, i.e., they employ natural languages.^24^ In this paper, we limit ourselves to sketch an informal ontological model.

At this point, though, following Rawls (2005) himself, a critic might point out that ontology is in fact incompatible with the kind of political liberalism that underlies our justice approach to gastrospaces.^25^ Yet, our decision to choose an ontological approach—among many other potential ones—to the study of gastrospaces rests on two related reasons which we think help us to eschew precisely that criticism. First, ontology relies on the most primitive “building bricks” of what there is. As such, it provides a more general and theoretically fundamental representation of reality than alternative approaches. With respect to gastrospaces, ontology provides a plurality of models for the same space without building on a specific ideology or conception of the good. It is therefore particularly in tune with the idea of liberal neutrality that underlies our Rawlsian justice framework and in keeping with the conception of justice that we employ in this paper, which is centered on the two moral powers rather than being grounded in any controversial conception of the good.

Second, and relatedly, while ontologists may often disagree in their conclusions, they share common standards of inquiry (Paul 2012; Hawley 2018). In this sense, ontology can be understood as a sort of *lingua franca* to be used for a multiplicity of purposes. Of course, as any other means of representation, ontology relies on specific linguistic and technological assumptions (e.g., using English or a formal language, employing a certain software, etc.) as well as on the social or perceptual bias that any individual ontologist inevitably has (e.g., preferring features that better match their own idea of what a gastrospace is). Yet all ontologists share a commitment to common methodological criteria which set the standard of their discipline. These include inference to the best explanation; semantic transparency; conceptual coherence; the use of conceptual or linguistic analysis; explicit disclosure of underlying conceptual assumptions; and a basic formal ontology that is substantially shared across all formal ontological endeavors (see Bonotti et al. 2022).

These considerations also serve to clarify why our choice of an ontological approach is not antagonistic to other scholarly approaches to the analysis of space, which could be employed to study gastrospaces—e.g., studies in anthropology (e.g., Low 2017), geography (Lefebvre 1996; McCann 2002), sociology (Gabrielson and Parady 2010), and philosophy of the city (King 2020). Rather, ontology can be used to generate bridges and dialogues across these various approaches, by providing shared means of representation and analysis while respecting the specificities of micro- or local analyses and histories. Granted, in this paper we can provide only the initial tools necessary to deliver a systematic ontological representation of gastrospaces and our analysis will limit itself to informal ontological modeling. But, as recent literature analyzing space by means of formal ontology suggests (e.g., Bateman et al. 2010; Chen et al. 2019; Boonstra & Rauws 2021), a formal model can be fruitfully developed and put to use once a suitable informal conceptual framework has been supplied.

Moving then forward with our outline of an ontological model of gastrospaces, three categories of entities strike us as most prominent: (i) the *types of agents* that act within such spaces; (ii) the *norms* underscoring these agents’ behaviors; and (iii) the *material conditions* of such spaces. We use each of these terms in a technical sense and, for this reason, it is worth considering them one at a time.

(i) *Agents*. These are entities that act in and set up the material conditions (e.g., furniture, walls, etc.) of gastrospaces, and that establish, abide by, or violate the social norms and institutional acts that regulate their functioning. Agents that populate gastrospaces are primarily humans; they can differ based on gender, sex, race, class, or other characteristics; they can also be categorized on the basis of the role thay accomplish in gastrospaces—e.g., customers, cooks, workers, dwellers, etc.—and they can be allocated to specific categories based on different criteria. However, besides humans, it seems reasonable that a broad ontological inventory of all possible things that can be regarded as agents should also include non-human agents. The list encompasses pets, wild animals, plants, ecosystems, and systems of microorganisms, among others. Take, for instance, the key role of yeasts in fermented foods, such as bread (Sariola 2021) and sake (Hey 2022). Such a broad conception of agency also aligns with recent literature in philosophy of biology, which has complexified the understanding of biological individuality (e.g., Godfrey-Smith 2013) and of agency^26^; it also resonates with ethical and legal theory, which regard as agents with politically relevant features large and small living systems (e.g., Celermajer et al. 2021), human-made machines (Allen et al. 2000), and possibly other entities too (e.g., Giaccardi et al. 2016).
(ii) *Norms*. Agents’ behavior in gastrospaces is paramountly governed by different sorts of norms. For a start, there are *social norms*, which emerge informally and are not codified by means of institutional acts, especially written ones (Bicchieri 2006). Furthermore, there are also *legal and institutional norms*, which are issued by a formal authority (e.g., the state, the local council, etc.) capable of imposing tangible penalties (e.g., fines). But it is important to also include *natural norms*, that is all those psychological or scientific regularities that may help to explain the behavior of agents—whether humans or non-humans—in a gastrospace.^27^ Recent literature in food psychology (e.g., Spence 2017), for example, has pointed out among others the importance of perceptual (e.g., color), linguistic (e.g., the name of a recipe on a menu), or design (e.g., the distribution of items on a buffet table) elements in norming the behavior of humans in a gastrospace.
(iii) *Material conditions*. This category includes all the physical features within or around a gastrospace, such as the physical size and dimensions of the space, its components, and their respective features, e.g., furniture (colors, dimensions, shape), outside/inside barriers, and other kinds of dividers (e.g., walls, doors, windows). These physical features also include the so-called mereotopological relations (i.e., parthood and connection relations) between different components of the physical space.

**Fig. 1:**
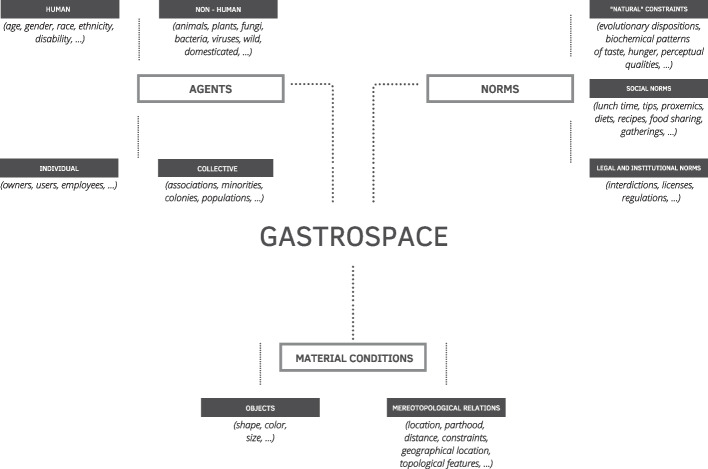
Illustration of the proposed ontological model for gastrospaces, which rests on three ontological categories: agents, norms, and material conditions

**Fig. 2:**
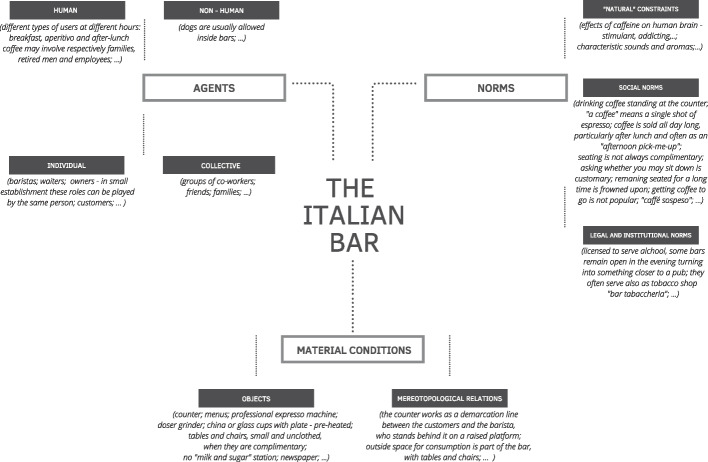
Illustration of an intended ontological model, representing a prototypical Italian bar. The model specifies key elements for each of the three ontological categories (agents, norms, and material conditions)

These three categories underlie a general model (Fig. 1) which provides an overview of the basic ontological components and entities of a gastrospace. However, the model can be differently realized, i.e., each category may comprise different specific entities and relations in alternative scenarios, serving different goals. That is, the membership conditions of a given category contextually vary based on a wide range of diverse factors, e.g., social structures, overarching norms, and specific epistemic, social, and pragmatic goals (see Borghini et al. 2020a, b for a similar analysis of food ontologies). We call these different realizations “intended (ontological) models of gastrospaces.” These intended models may be more or less general: they may concern the realization of a class of places (e.g., restaurants) or a specific instantiation of a place (this specific restaurant I am designing or talking about) (Fig. 2).

### Gastrospaces: Key Features

The purpose of our general ontological model and of different intended models is to map out and make explicit all the constituents of gastrospaces. These models, however, are far from capturing the everyday representations of such spaces that guide social interactions. In fact, when people approach gastrospaces, they do so with specific experiences in mind (e.g., gathering with friends). In the account offered by Shapin (2020), for instance, diners at Buck’s (a restaurant in Woodside, California, whose regulars are Silicon Valley entrepreneurs and venture capitalists) seek out an informal and intimate place, “designed (partly intentionally, partly accidentally) to be just the right setting for capital-meets-technoscientific-entrepreneurship” (2020: 336). Another neat case in point is offered by White’s (2012) analysis of coffee houses in Japan, which shows their multiple functions—from places where migrants could “understand a big city quickly” (2012: 139) to refuges from the urban pressure where “being alone is what is a premium good, not being together” (2012: 20), or even places where employees who frequent them can be their “other selves,” communicating ideas and feelings barely shareable at work, and even at home (2012: 159).^28^

Our contention is that these and countless other experiences are typically enabled by certain *(meta-)features* of gastrospaces: these are abstract qualities of such spaces (e.g., the spatial arrangement of chairs and tables in a dining room) which can be linked to their functions (e.g., providing exclusive conversation opportunities for diners) or to the physical realization of certain political ideas (e.g., an ideology such as Marxism),^29^ aesthetic concepts (e.g., harmony or elegance), or other abstract goals.^30^ By way of illustration, in this study we focus on five prominent (meta-)features: (i) temporal and (ii) spatial flexibility; (iii) versatility; (iv) power distribution and authority; and (v) degree and type of social exchange.^31^

These (meta-)features are highly abstract and can be realized jointly or separately via diverse intended models of gastrospaces, by combining the components of various ontological categories in disparate ways.^32^ Furthermore, the (meta-)features seem to present a number of shared traits. First, they come in degrees, i.e., they can be more or less present in a gastrospace; second, none of these features is positive or negative per se, i.e., their presence or absence can either advance or hinder the realization of certain justice goals; third, each feature can be realized in multiple ways by different kinds of entities; fourth, these features are interrelated.

(i) *Temporal flexibility.* A gastrospace can be more or less flexible in terms of time organization and management. Its temporality affects and is affected by the temporalities of the specific entities and agents that populate it. One example are opening hours, which can be determined by the law or by other social norms as well as by the personal inclination of relevant agents, e.g., the owner or the guests. Additional temporal constraints that affect flexibility may include seating times and temporal constraints associated with food.
(ii) *Spatial Flexibility.* The spatial organization of a gastrospace can be more or less static. For example, gastrospaces may or may not have adjustable furniture that can be moved when needed; they may or may not enable a high variety of interactions with(in) their environment (e.g., a kitchen may have a folding dining table; a restaurant may allow customers to sit or just stand, and to do so indoor or outdoor) as well as with(in) the broader surroundings (e.g., depending on whether they are well connected with other different places). Furthermore, gastrospaces may accommodate to different degrees the special requirements of certain agents, such as customers in wheelchairs.
(iii) *Versatility.* The versatility of a gastrospace consists in its ability to perform and be used for different functions. Some gastrospaces can perform multiple functions at the same time, without significant changes to their material conditions (e.g., spaces where you can have a coffee or a cocktail at the same hour, sitting at the same table). Conversely, others realize their versatility by modifying some of their ontological components (e.g., material conditions) according to a schedule and managing the space consequently (e.g., turning a dining room into a ballroom after 9 pm thanks to the presence of sufficient space and movable tables).
(iv) *Power distribution and authority.* Gastrospaces generally involve power-based role distinctions between the various agents that act within or around them. These role distinctions provide each agent (or type of agents) with specific powers that can affect material conditions, norms, and other agents in that space—e.g., the chef who has the power to determine the menu. Furthermore, interactions and exchanges between different agents in a gastrospace are generally regulated by an authority. This authority can be *internal*—i.e., settled and handled by insiders (e.g., the owner), and its power limited and only exercised upon the people who frequent that place—or *external*—i.e., it exercises its power more widely, including in places beyond the one at stake, and it is held by a recognized institution (e.g., the local health department).
(v) *Degree and type of social exchange.* An additional feature of gastrospaces is the degree and type of social interactions that they allow and encourage. Some gastrospaces allow a high level of interaction among people from diverse backgrounds and identities (e.g., racial, religious, gender, socio-economic, etc.). Others, instead, favor interaction between people with the same background or identity, like the aforementioned example of Buck’s restaurant, which women (as well as Hispanics and African Americans) hardly frequent (Shapin 2020). Still, others may discourage interaction among patrons and instead promote solo dining experiences, as in the case of Ichiran Restaurant in New York.^33^

## Conclusion: From Analysis to Intervention

In this paper, we have combined political philosophy and ontology to provide a novel normative framework for evaluating gastrospaces. First, we illustrated the value of gastrospaces from the perspective of political philosophy, by explaining how such spaces can help individuals to develop and exercise their two moral powers. We then complemented this analysis with a conceptual framework that includes an informal ontological model and five (meta-)features. What we have offered is, of course, an abridged version of a broader theoretical perspective, which purports to capture the wide variety of gastrospaces and their functions in our social and political life.

Our framework has potential practical implications too. To understand why, consider, for example, the (meta-)features that we identified in the previous section. These offer a map to guide justice-oriented interventions, by representing *the potential dimensions along which* the design or modification of gastrospaces may be carried out in order to promote, and potentially address, any obstacles to the development and exercise of the two moral powers. But what would these interventions look like? While we cannot address this question in depth here, we shall conclude by pointing out some directions an answer could take.

At the *institutional level*, gastrospaces can be regulated by various legal or political authorities (e.g., state, council). Relevant measures may include, for example, laws that regulate the opening hours of cafes and restaurants, as well as those that establish whether, when, and how food (and drink) can be consumed, and whether certain gastrospaces can discriminate against certain types of customers. Consider, for example, the case of gay bars discussed earlier. The opportunity that gay people have to meet in these gastrospaces *qua* members of the same group is precisely what renders those spaces particularly valuable for them. In such spaces, gay people can develop and exercise their second moral power by expressing their identities and jointly pursuing their conceptions of the good (e.g., by enjoying certain types of foods) as well as advance justice goals associated with the first moral power (e.g., creating coalitions of support for LGBTQ + rights). Ensuring that this and other vulnerable and marginalized groups remain in control of certain gastrospaces—thus de facto limiting the latter’s degree of social exchange—would therefore seem to be central to those group members’ ability to develop and exercise their moral powers, and this may require allowing them to exclude out-group members, thus exempting them from anti-discrimination laws that would normally apply to other gastrospaces.

But interventions can also be implemented by *gastrospace keepers*, i.e., those who own or manage, even temporarily, gastrospaces. Our ontological framework can help those actors not only to identify instances of injustice concerning such spaces but also to understand how they could voluntarily modify their establishments in ways that may better align with certain justice goals. For example, in response to criticisms of racist dress codes in some restaurants in the US, one commentator (Saxena 2020) pointed out that “restaurants are facing a unique opportunity to change how business is done, and an imperative to make things equitable. One small step in this direction is to abolish dress codes.” While one might argue that in this case legal/institutional interventions would be more appropriate, given the serious nature of the injustice at stake, in other cases restaurateurs may indeed be the best implementers of interventions aimed at (re)designing gastrospaces, setting aside the risk of excessive invasiveness that legal interventions may often carry with them.

Finally, our framework can also be useful for *gastrospace users*, from restaurant customers to beachgoers and those who enjoy eating in public parks or in their home kitchen. For all these different types of agents, knowing whether and to what extent any of the five (meta-)features is present in a gastrospace can be crucial for their ability to develop and exercise their moral powers. Our framework can provide gastrospace users with a clear understanding of what to look for when they have to decide in which gastrospaces they can best develop and exercise their moral powers.

This overview of potential areas of gastrospace interventions is admittedly sketchy and a more systematic analysis cannot be accommodated within the limited space of this paper. However, we hope that our conceptual and normative analysis of gastrospaces will open up a new research agenda, which critically re-evaluates the role and function of gastrospaces in our daily lives.

## Footnotes

1 In keeping with the philosophical literature on food, we employ terms such as “eating” and “food” to also cover all the activities generally related to drinking and the relevant spaces where those activities occur, such as cafes, pubs and bars.

2 While there is an emerging literature on food justice in political philosophy (e.g., Barnhill and Bonotti 2022; Bonotti and Ceva 2015, 2016) as well as a growing body of work on the philosophy of the city that examines urban life and public spaces in relation to justice and democracy (e.g., Bell and de Shalit 2014; de Shalit 2018; Kohn 2016; Parkinson 2014), to our knowledge no author, within the political philosophical domain, has so far endeavored to examine the justice dimensions of gastrospaces.

3 We follow in the footsteps of other studies concerning the ontology of food and eating practices, such as Borghini and Engisch (2022); Borghini et al. (2021, 2022).

4 See Bascuñan-Wiley et al. (2022) for an ethnographic survey of this phenomenon.

5 Although we have chosen a Rawlsian framework because we consider it sufficiently neutral to accommodate a variety of conceptions of the good, we acknowledge that alternative approaches to the justice of gastrospaces could be developed based on different conceptions of justice. For example, one could imagine a communitarian approach to gastrospaces, centered on the protection and promotion of certain traditional cultural and religious values, or a libertarian approach that aims to minimize government interference with people’s freedom in and around gastrospaces. Based on those (or other) justice perspectives, what counts as justice and injustice in the context of gastrospaces may look quite different from what we consider justice and injustice in our analysis. While we do not have the space to discuss these alternative justice approaches here, we welcome future analyses along these lines.

6 An important question arises in relation to this point: do institutions or other agents bear a moral duty to fulfill transient or “invisible” individuals’ moral powers? Although these are important questions, we set them aside for future studies. See, for instance, Chez (2011).

7 The social gastronomy movement has already emphasized the contribution of gastrospaces to achieve and preserve social goods; also, Borghini and Baldini (2022) and Borghini and Piras (2022) have shown that, in some instances, gastrospaces can become a prominent form of public art that serves to preserve societal memory, to foster social emancipation, or to enact a protest. Gastrospaces such as *Conflict Kitchen* in Pittsburgh, for instance, utilize certain occasions to re-enact political issues by offering dishes originating in countries in conflict with the United States; see https://www.conflictkitchen.org and Flammang (2016: 178-184). In our study, however, we adopt an approach more distinctively grounded in political philosophy.

8 See, for instance, the contribution made by the synagogue canteen in Babruisk to rebuilding local social and identity relationships (https://voyages.eurasia.undp.org/in-belarus-a-synagogue-spurs-a-towns-rebirth/).

9 See also Borghini and Baldini (2022) on how the preparation and consumption of specific foods can enable and empower artistry and creativity during daily and working activities.

10 See Lukito and Xenia (2017). To illustrate further, consider food centers that foster labor inclusion among immigrants, such as the Food Enterprise Center (https://dalia-mortada-4h8p.squarespace.com/food-enterprise-center) and Foodhini (https://foodhini.com/pages/mission).

11 Self-respect can further be enhanced when individuals can see their culinary culture socially recognized via gastrospaces, e.g., when immigrants see that their “ethnic” restaurants, grocery stores, or similar outlets are allowed or even celebrated.

12 In keeping with Zuolo (2020), we acknowledge that while a commitment to animal welfare is widely shared in diverse societies—and, therefore, a legitimate concern of justice—there can be disagreement as to whether selected issues in animal ethics, such as animal rights to life or liberty, should be central to a political conception of justice in these societies.

13 See, for example, Harris’s (2017) account of this new phenomenon in the USA.

14 https://communitykitchens.org.au/philosophy/

15 Social primary goods include liberties, income, wealth, and social opportunities (Cordelli 2015: 94).

16 A good example of a gastrospace trying to address this problem is South Philly Barbacoa in Philadelphia, run by chef Cristina Martínez, whose opening hours (5am-3 pm) are deliberately designed to fit many immigrant workers’ schedules. See https://generocity.org/philly/2018/09/11/the-immigrant-activists-of-south-philly-barbacoa-will-be-featured-on-netflixs-chefs-table-cristina-martinez-benjamin-miller/ (Accessed 5 December 2022).

17 Although this is a rare occurrence, some political philosophers have engaged in this kind of analysis, by coupling normative theories of justice with precise descriptions of the tools and agents necessary to implement them. See, for instance, the accurate account of capabilities provided by Sen (1985).

18 See among others Erickson (2004); Sutton (2007); Sandiford (2019).

19 Other conceptualizations of third places are developed by Bhabha (1994) and Soja (1996) who provide entirely different frameworks while adopting the same terminological label. We do not engage with their work here for the sake of brevity.

20 This approach would follow in the footsteps of Crowther (2013: 177-206).

21 For instance, Purnell (2015) tried to widen the scope of Oldenburg’s model in order to also include private spaces. Dolley and Bosnan (2019) collect several critical essays which aim to amend Oldenburg’s original model.

22 For instance, Ichiran Restaurant in New York is renowned for offering a unique solo dining experience, which is unusual for Western diners. See https://www.forbes.com/sites/akikokatayama/2019/04/21/the-solo-dining-ramen-experience-at-ichiran-demonstrates-its-global-appeal/. (Accessed 5 December 2022).

23 See for instance, formal geospatial ontologies (Casati et al. 1998) as well as ontologies for biomedical research or for biological taxonomies (Lean 2021). Some of these ontologies have been used as basic taxonomies of computer-based information systems for storing and sharing data.

24 Similar ontological exercises have been successfully carried out for other domains of everyday life (for an analysis of this notion see Saito 2017), such as geography (Smith and Mark 2003), cities (Varzi 2021), and food (Borghini and Piras 2021).

25 True, Rawls refers to “metaphysics” rather than “ontology” in his book *Political Liberalism* (2002). However, we assume that Rawls is in fact also referring to ontology (as we use the term in this paper). Indeed, as Rosenthal points out, “[Rawls’s] understanding of the term [metaphysics] includes features central to the notion of ontology used by the current ontological turn. For example, Rawls says that ‘metaphysical claims’ may involve a thesis about agency: the ‘nature of persons as moral or political agents’ (1996: 29). Rawls also uses the term metaphysical for general views about the constitutive elements of social reality, such as Habermas’ ontology of communicative action in the lifeworld (1996: 378–379). This [i.e. Rosenthal’s] essay [therefore] uses the term ontological instead of metaphysical, except in the citations” (2019: 256, footnote 3).

26 See, e.g., Pradeu et al. (2016) for a definition of viruses as agents.

27 As Thomson (2008, chapter XII) puts it, norms can be understood as directives that “include judgments that are not about people,” as when we say, for instance, that “[a] toaster ought to toast toastables—bread, bagels, frozen waffles, and the like […] [t]he pancreas ought to secrete digestive enzymes” (2008: 207). The understanding of normativity underlying these uses of “ought,” Thomson explains, expresses what specific entities, in specified contexts, are called on or required to do either to follow their nature (e.g., a restaurant ought to serve food in order to be a restaurant) or to reach specific goals (e.g., a virus ought to be infectious in order to replicate itself). Moreover, as McAdams (2001: 2735) puts it, a norm “is a regularity of behavior among a population of individuals, where the regularity is at least partly sustained by the fact that at least most individuals approve conformity to the regularity and/or disapprove nonconformity.” And, as Lorini (2022) argues, different senses of normativity can also be applied to the social behavior of non-human animals, to the extent that we should set up a new discipline, “the ethology of normativity.”.

28 Additional examples include Ray (2014), who studies the role of immigrant restaurateurs in constructing new semi-public spaces and their contribution to the culture and economy of American cities, and Lee (2019), who argues that “ethnic” restaurants provide immigrant citizens with a new set of rights directly related to the communal consumption of food.

29 Consider, for instance, the different ways in which urban planners have tried to translate Marxist ideals into urban plans (Knox 1987; Holgersen 2020) and various forms of architecture (Kambuj 1980).

30 We may help ourselves here by means of a comparison with architectural typologies, where the social function of buildings varies independently of a specific architectural typology. A well-known case in point is that of synagogues, which notoriously lack a codified typology. Here, the typical functions of a synagogue (e.g., providing a place for worship and performing community ceremonies) are realized by different ontological models in different contexts, e.g., a hidden room inside a house, an outside space, a stand-alone building, etc.

31 We do not claim that this list of (meta-)features is exhaustive and we do not exclude that in specific instances other non-key features may also be present and be employed to integrate the framework that we outline here. However, for the sake of space we set these issues aside here.

32 This idea is not new in philosophy. Just think of aesthetic properties that can be differently realized by different artworks (Sibley 2001) as well as the idea of “right” which can be instantiated in different actions (Ross 1930).

33 See footnote 21.

## References

[CR1] Allen C, Varner G, Zinser J (2000). Prolegomena to any future artificial moral agent. J Exp Theor Artif Intell.

[CR2] Allport GW (1954). The nature of prejudice.

[CR3] Ammar A, Brach M, Trabelsi K, Chtourou H, Boukhris O, Masmoudi L, Bouaziz B, Bentlage E, How D, Ahmed M, Müller P, Müller N, Aloui A, Hammouda O, Paineiras-Domingos LL, Braakman-Jansen A, Wrede C, Bastoni S, Pernambuco CS, … Hoekelmann A (2020) Effects of COVID-19 home confinement on eating behaviour and physical activity: results of the ECLB-COVID19 international online survey. Nutrients 12(6):1583. 10.3390/nu12061583 10.3390/nu12061583 PMC7352706 32481594

[CR4] Arp R, Smith B, Spear AD (2015). Building ontologies with basic formal ontology.

[CR5] Barbieri J, Riva F, Colombo E (2017). Cooking in refugee camps and informal settlements: A review of available technologies and impacts on the socio-economic and environmental perspective. Sustainable Energy Technol Assess.

[CR6] Barnhill A, Bonotti M (2022). Healthy eating policy and political philosophy. A public reason approach.

[CR7] Barnhill A, King KF, Kass N, Faden R (2014). The value of unhealthy eating and the ethics of healthy eating policies. Kennedy Inst Ethics J.

[CR8] Bascuñan-Wiley N, DeSoucey M, Fine GA (2022). Convivial quarantines: Cultivating co-presence at a distance. Qual Sociol.

[CR9] Bateman JA, Hois J, Ross R, Tenbrink T (2010). A linguistic ontology of space for natural language processing. Artif Intell.

[CR10] Bell D A, de Shalit A (2014) The spirit of cities. Why the identity of a city matters in a global age. Princeton University Press, Princeton

[CR11] Bhabha HK (1994). The location of culture.

[CR12] Bicchieri C (2006). The grammar of society. The nature and dynamics of social norms.

[CR13] Boonstra B, Rauws W (2021) Ontological diversity in urban self-organization: Complexity, critical realism and post-structuralism. Plan Theory 1473095221992392.10.1177/1473095221992392

[CR14] Bonotti M, Ceva E (eds) (2015) Special issue: Symposium on the political philosophy of food policies, part I: Justice, legitimacy, and rights. J Soc Philos 46(4)

[CR15] Bonotti M, Ceva E (eds) (2016) Special issue: Symposium on the political philosophy of food policies, part II: Democracy, freedom, and paternalism. J Soc Philos 47(1)

[CR16] Bonotti M, Borghini A, Piras N, Serini B (2022). Learning from covid-19: Public justification and the ontology of everyday life. Soc Theory Pract.

[CR17] Bonotti M, Zech S (2021). Recovering civility during Covid-19.

[CR18] Borghini A, Baldini A (2022). Cooking and dining as forms of public art. Food, Culture & Society.

[CR19] Borghini A, Engisch P (eds) (2022) A philosophy of recipes. Making, experiencing, and valuing. Bloomsbury, London

[CR20] Borghini A, Piazza T, Mogensen L, Forsey J (2019). The aesthetic properties of wine. On taste: Aesthetic exchanges.

[CR21] Borghini A, Piras N (2021) On interpreting something as food. Food Ethics 6(1). 10.1007/s41055-020-00082-5

[CR22] Borghini A, Piras N (2022) Eating local as public art. Aisthesis 15(1):15–27. 10.36253/Aisthesis-13449

[CR23] Borghini A, Piras N, Serini B (2020). Ontological frameworks for food utopias. Riv Estetica.

[CR24] Borghini A, Piras N, Serini B (2020b) A gradient framework for wild foods. Studies in History and Philosophy of Science Part C: Studies in History and Philosophy of Biological and Biomedical Sciences 101293.10.1016/j.shpsc.2020.101293 10.1016/j.shpsc.2020.101293 32713789

[CR25] Borghini A, Piras N, Serini B (2021). Defective food concepts. Synthese.

[CR26] Borghini A, Piras N, Serini B (2022). Eating local: A philosophical toolbox. Philos Q.

[CR27] Burnett J (2004). England eats out: A social history of eating out from 1830 to the present.

[CR28] Carbonara E, Parisi F (2017). Law and social norms. The Oxford handbook of law and economics.

[CR29] Casati R, Smith B, Varzi AC, Guarino N (1998). Ontological tools for geographic representation. Formal ontology in information systems.

[CR30] Celermajer D, Schlosberg D, Rickards L, Stewart-Harawira M, Thaler M, Tschakert P (2021). Multispecies justice. Theories, challenges, and a research agenda for environmental politics. Environ Polit.

[CR31] Chen X, Kim T W, Chen J, Xue B, Jeong W (2019) Ontology-based representations of user activity and flexible space information: Towards an automated space-use analysis in buildings. Adv Civil Eng e3690419. 10.1155/2019/3690419

[CR32] Cordelli C (2015). Justice as fairness and relational resources. J Polit Philos.

[CR33] de Shalit A (2018). Cities and immigration. Political and moral dilemmas in the new era of migration.

[CR34] Dolley J, Bosnan C (2019). Rethinking third places: Informal public spaces and community building.

[CR35] Dooley DM, Griffiths EJ, Gosal GS, Buttigieg PL, Hoehndorf R, Lange MC, Schriml LM, Brinkman FSL, Hsiao WWL (2018). Foodon: A harmonized food ontology to increase global food traceability, quality control and data integration. NPJ Science of Food.

[CR36] Chapple-Sokol S (2013). Culinary diplomacy: Breaking bread to win hearts and minds. Hague J Dipl.

[CR37] Chez K (2011). Popular ethnic food guides as auto/ethnographic project: The multicultural and gender politics of urban culinary tourism. J Am Cult.

[CR38] Crowther G (2013). Eating culture. An anthropological guide to food.

[CR39] Erickson K (2004). Bodies at work: Performing service in american restaurants. Space Cult.

[CR40] Fainstein S (2010). The just city.

[CR41] Flammang JA (2016). Table talk: building democracy one meal at a time.

[CR42] Gabrielson T, Parady K (2010). Corporeal citizenship: Rethinking green citizenship through the body. Environmental Politics.

[CR43] Garcia ME (2021). Gastropolitics and the specter of race. Stories of capital, culture, and coloniality in Peru.

[CR44] Gibson S, Molz JG (2016). Mobilizing hospitality. The ethics of social relations in a mobile world.

[CR45] Giaccardi E, Speed C, Cila N, Caldwell ML, Smith RC, Tang Vangkilde K, Kjaersgaard MG, Otto T, Halse J, Binder T (2016). Things as co-ethnographers: Implications of a thing perspective for design and anthropology. Design anthropological futures.

[CR46] Godfrey-Smith P, Bouchard F, Huneman P (2013). Darwinian individuals. From groups to individuals evolution and emerging individuality.

[CR47] Gopnik A (2018) What cafés did for liberalism. The New Yorker. URL: https://www.newyorker.com/magazine/2018/12/24/what-cafes-did-for-liberalism. Accessed 5 Dec 2022

[CR48] Groff RP (2013). Ontology revisited. Metaphysics in social and political philosophy.

[CR49] Guttmann A, Thompson DF (2004). Why deliberative democracy?.

[CR50] Habermas J (1989). The structural transformation of the public sphere. An inquiry into a category of bourgeois society.

[CR51] Harris J (2017) The restaurant that charges different prices for the same meal based on neighborhood income is expanding, Los Angeles Times. https://www.latimes.com/food/dailydish/la-fo-restaurant-news-20170228-story.html. Accessed 10 Jan 2023

[CR52] Haslanger S (2012). Resisting reality. Social construction and social critique.

[CR53] Hawley K (2018). Social science as a guide to social metaphysics?. J Gen Philos Sci.

[CR54] Henderson S, Coleman D, Danze E, Henderson C, Henderson S (1996). A revolution in the woman’s sphere: Grete Lihotsky and the Frankfurt kitchen. Architecture and Feminism.

[CR55] Hessler M, Oldenzile R, Zachman K (2009). The Frankfurt kitchen: The model of modernity and the “madness” of traditional users, 1926 to 1933. Cold war kitchen: Americanization, technology and European users.

[CR56] Hey M, Borghini A, Engisch P (2022). On attunement: fermentation, feminist ethics, and relationality in sake-making practices. A philosophy of recipes. Making, experiencing, and valuing.

[CR57] Holgersen S (2020). On spatial planning and marxism: looking back, going forward. Antipode.

[CR58] Isin E, Shachar A, Bauböck R, Bloemraad I, Vink M (2017). Performative citizenship. The Oxford handbook of citizenship.

[CR59] Jacobs M, Scholliers P (2003). Eating out in Europe: Picnics, gourmet dining and snacks since the late eighteenth century.

[CR60] Janner M, Narasimhan K, Barzilay R (2018). Representation learning for grounded spatial reasoning. Trans Assoc Comput Linguistics.

[CR61] Jeffres LW, Bracken CC, Jian G, Casey MF (2009). The impact of third places on community quality of life. Appl Res Qual Life.

[CR62] Kambuj DM (1980). Marxist position in aesthetics of architecture. Social Scientist.

[CR63] Ketchum A (2018) Serving up revolution: feminist restaurants, cafés, and coffeehouses in the United States and Canada from 1972–1989. Ph.D. Dissertation. McGill University

[CR64] Khan AZ, Moulaert F, Schreurs J (2013). Epistemology of space: Exploring relational perspectives in planning, urbanism, and architecture. Int Plan Stud.

[CR65] King L, Meagher MS, Noll S, Biehl JS (2020). Henri Lefevbre and the right to the city. The Routledge handbook of the philosophy of the city.

[CR66] Knox P L (1987) The social production of the built environment. architects, architecture and the post-modern city. Prog Hum Geogr 11(3):354–377. 10.1177/030913258701100303

[CR67] Kohn M (2016). Death and life of the urban Commonwealth.

[CR68] Lean OM (2021). Are bio-ontologies metaphysical theories?. Synthese.

[CR69] Lee C T (2019) Improvising “nonexistent rights”: Immigrants, ethnic restaurants, and corporeal citizenship in suburban California. Social Inclusion 7(4): 79–89. 10.17645/si.v7i4.2305

[CR70] Lefebvre H (1996). Writing on cities.

[CR71] List C, Pettit P (2011) Group agency. The possibility, design, and status of corporate agents. Oxford University Press, Oxford

[CR72] Longhurst R, Johnston L, Ho E (2009). A visceral approach: cooking ‘at home’ with migrant women in Hamilton New Zealand. Trans Inst Br Geogr.

[CR73] Lorini G (2022). Animal Norms: An investigation of normativity in the non-human social world. Law, Culture and the Humanities.

[CR74] Low, S (2017) Spatializing culture. The ethnography of space and place. Routledge, London

[CR75] Lukito YN, Xenia AP (2017). Café as third place and the creation of a unique space of interaction in UI Campus. IOP Conference Series: Earth and Environmental Science.

[CR76] Marovelli B (2019). Cooking and eating together in london: food sharing initiatives as collective spaces of encounter. Geoforum.

[CR77] McAdams, R. H. (2001). Conventions and Norms: Philosophical Aspects. In Smelser N J, Baltes P D (eds) International Encyclopedia of the Social & Behavioral Sciences*.* Elsevier, Amsterdam, pp 2735–2741. 10.1016/B0-08-043076-7/01002-0

[CR78] McCann EJ (2002). Space, citizenship, and the right to the city: A brief overview. GeoJournal.

[CR79] Meah A, Jackson P (2016). Re-imagining the kitchen as a site of memory. Soc Cult Geogr.

[CR80] Mulvaney-Day N, Womack CA (2009). Obesity, identity and community: Leveraging social networks for behavior change in public health. Public Health Ethics.

[CR81] Navarro-Dols J, González-Pernía JL (2020). Gastronomy as a real agent of social change. International Journal of Gastronomy and Food Science.

[CR82] Oldenburg R (1999) The great good place. Paragon House, Manchester

[CR83] Parkinson JR (2014). Democracy and public space.

[CR84] Paul LA (2012). Metaphysics as modeling: The handmaiden’s tale. Philos Stud.

[CR85] Pettigrew TF (1998). Intergroup contact theory. Annu Rev Psychol.

[CR86] Phan T-T, Labhart F, Muralidhar S, Gatica-Perez D (2020) Understanding heavy drinking at night through smartphone sensing and active human engagement. Proceedings of the 14th EAI International Conference on Pervasive Computing Technologies for Healthcare, 211–222. 10.1145/3421937.3421992

[CR87] Pradeu T, Kostyrka G, Dupré J (2016). Understanding viruses: Philosophical investigations. Studies in History and Philosophy of Science Part c: Studies in History and Philosophy of Biological and Biomedical Sciences.

[CR88] Purnell D (2015). Expanding Oldenburg: Homes as third places. J Place Manag Dev.

[CR89] Rawls J (1999). A Theory of justice.

[CR90] Rawls J (2005). Political liberalism.

[CR91] Rawson K, Shore E (2019). Dining out: A global history of restaurants.

[CR92] Ray K (2014). The immigrant restaurateur and the American city: Taste, toil, and the politics of inhabitation. Soc Res.

[CR93] Rosenthal I (2019). Ontology and political theory: A critical encounter between Rawls and Foucault. Eur J Polit Theo.

[CR94] Ross WD (1930). The right and the good.

[CR95] Rucks-Ahidiana Z, Bierbaum AH (2015). Qualitative spaces: Integrating spatial analysis for a mixed methods approach. Int J Qual Methods.

[CR96] Saito Y (2017). Aesthetics of the familiar. Everyday life and the world-making.

[CR97] Sandiford PJ (2019). The third place as an evolving concept for hospitality researchers and managers. J Hosp Tour Res.

[CR98] Sariola S (2021). Fermentation in post-antibiotic worlds: Tuning in to sourdough workshops in finland. Curr Anthropol.

[CR99] Saxena J (2020) Restaurant dress codes frequently target black customers. It’s past time for them to go. The Eater. https://www.eater.com/21546024/restaurant-dress-code-discrimination-prejudice-history. Accessed 5 Dec 2022

[CR100] Shaftoe H (2015). Convivial urban spaces. Creating effective public places.

[CR101] Shapin S (2020). breakfast at Buck’s: Informality, intimacy, and innovation in Silicon Valley. Osiris.

[CR102] Sen A (1985). Commodities and capabilities.

[CR103] Sibley F (2001). Approach to aesthetics. Collected papers on philosophical aesthetics.

[CR104] Sisson P (2016) How gay bars have been a building block of the LGBTQ community. Curbed. https://archive.curbed.com/2016/6/17/11963066/gay-bar-history-stonewall-pulse-lgbtq. Accessed 10 Jan 2023

[CR105] Smith B, Kanzian C (2007). On place and space: The ontology of Eruv. Cultures: Conflict - analysis - dialogue.

[CR106] Smith B, Mark DM (2003). Do mountains exist? Towards an ontology of landforms. Environ Plann B Plann Des.

[CR107] Smith G, Setälä M, Bächtiger A, Dryzek JS, Mansbridge J, Warren M (2018). Mini-publics and deliberative democracy. The Oxford handbook of deliberative democracy.

[CR108] Soja E (1996). Thirdspace: Journeys to Los Angeles and other real-and-imagined places.

[CR109] Spence C (2017). Gastrophysics. The new science of eating.

[CR110] Supski S (2006). ‘It was another skin’: The kitchen as home for Australian post-war immigrant women. Gend Place Cult.

[CR111] Sutton D, Beriss D, Sutton D (2007). Tipping: An anthropological meditation. The Restaurants Book. Ethnographies of Where We Eat.

[CR112] Thomson JJ (2008). Normativity.

[CR113] Varzi AC (2021). What is a city?. Topoi.

[CR114] Warde A, Martens L (2000). Eating out. Social differentiation, consumption and pleasure.

[CR115] Warde A, Whillans J, Paddock J (2019). The allure of variety: Eating out in three English cities, 2015. Poetics.

[CR116] Wexler M, Oberlander J (2017). The shifting discourse on third places: Ideological implications. J Ideol.

[CR117] White M (2012). Coffee life in Japan.

[CR118] Zuolo F (2020). Animals, political liberalism and public reason.

